# The impact of infectious diseases on personality traits - comparative study on HIV versus hepatitis B and C

**DOI:** 10.1186/1471-2334-14-S4-P15

**Published:** 2014-05-29

**Authors:** Carmen Manciuc, Maria Alexandra Largu, Andrei Vâță, Cristina Nicolau, Liviu Jany Prisăcariu, Daniela Stoica, Carmen Dorobăț

**Affiliations:** 1 Infectious Diseases Hospital “Sf Parascheva”, Iaşi, Romania

## 

Personality traits are in constant dynamics and they are influenced by significant events in the life of the person. The study aims to evaluate chronic infectious diseases patients (HIV, HBV, HCV positive) from a psychological perspective, for a period of 9 months, January-September 2013.

We evaluated 52 HIV-positive patients in evidence at the Iaşi Regional Center, and 48 patients diagnosed with chronic hepatitis B or C. They were evaluated using the A. P. Questionnaire to detect accentuated personalities, developed by Dr. H. Schmieschek.

Most patients enrolled were female (51%). The median age was 24.3 years for the HIV-positive lot and 34 for the HBV/HCV lot; 34% of them came from rural areas. Average schooling level was 10 classes; 38% came from broken families or foster care; 15.3% of the HIV/AIDS patients and 62.5% of HBV/HCV patients had a stable job. All of the HIV positive patients were in active therapy, polyexperimented. All of the HBV/HCV infected patients were in active therapy with pegylated-interferon. The A. P. Questionnaire found in HIV infected patients: 90% emotivity, 85% anxiety, and 65% dysthymia (alternative episodes of exaltation and depression). In the case of HBV and HCV infected patients, 60% scored high anxiety, other accentuated personality traits being distributed according to the specifics of each patient.

The dominant personality traits that are accentuated when faced with an infectious disease have certain specific trends depending on the disease, duration of therapy and psycho-social impact of the diagnosis.

